# Supplementary material for: Fundamental Characterization of Antibody Fusion-Single-Chain TNF Recombinant Proteins Directed against Costimulatory TNF Receptors Expressed by T-Lymphocytes
Source: Cells. 2023 Jun 9;12(12):1596. doi: 10.3390/cells12121596 (PMC10297085; doi:10.3390/cells12121596)
Supplement: Supplementary file 1 [file cells-12-01596-s001.zip › cells-2404242-supplementary.pdf]

Figure S1

A **hIL-2Rγ signal sequence-Fc (hinge, CH2 and CH3)-PA**

(EcoRI)-(START)-hIL-2Rγ signal sequence-(BamHI)-Fc (hinge, CH2 and CH3)-PA tag

GAATTCTCTAGAGATCCCTCGACCTCGAGATCCATCGGGCCCCCCCTCGAGGTCGACAGCAAGCGCCATGTTGAAGCCATCATTACCATTCACATCCCTCTTATTCCTGCAGCTGCCCCTGCTGGGAG
TGGGGCTGAACACGACAATTCTGACGCCCGGTAAGTATAGGATCCACTAGTTCTATCCTGCCTGGACGCATCCCGGCTATGCAGTCCCAGTCCAGGGCAGCAAGGCAGGCCCCGTCTGCCTCTTCACC
CGGAGGCCTCTGCCCGCCCCACTCATGCTCAGGGAGAGGGTCTTCTGGCTTTTTCCCCAGGCTCTGGGCAGGCACAGGCTAGGTGCCCCTAACCCAGGCCCTGCACACAAAGGGGCAGGTGCTGGGCT
CAGACCTGCCAAGAGCCATATCCGGGAGGACCCTGCCCCTGACCTAAGCCCACCCCAAAGGCCAAACTCTCCACTCCCTCAGCTCGGACACCTTCTCTCCTCCCAGATTCCAGTAACTCCCAATCTTC
TCTCTGCAGAGCCCAAATCTTGTGACAAAACTCACACATGCCCACCGTGCCCAGGTAAGCCAGCCCAGGCCTCGCCCTCCAGCTCAAGGCGGGACAGGTGCCCTAGAGTAGCCTGCATCCAGGGACAG
GCCCCAGCCGGGTGCTGACACGTCCACCTCCATCTCTTCCTCAGCACCTGAACTCCTGGGGGGACCGTCAGTCTTCCTCTTCCCCCCAAAACCCAAGGACACCCTCATGATCTCCCGGACCCCTGAGG
TCACATGCGTGGTGGTGGACGTGAGCCACGAAGACCCTGAGGTCAAGTTCAACTGGTACGTGGACGGCGTGGAGGTGCATAATGCCAAGACAAAGCCGCGGGAGGAGCAGTACAACAGCACGTACCGT
GTGGTCAGCGTCCTCACCGTCCTGCACCAGGACTGGCTGAATGGCAAGGAGTACAAGTGCAAGGTCTCCAACAAAGCCCTCCCAGCCCCCATCGAGAAAACCATCTCCAAAGCCAAAGGTGGGACCCG
TGGGGTGCGAGGGCCACATGGACAGAGGCCGGCTCGGCCCACCCTCTGCCCTGAGAGTGACCGCTGTACCAACCTCTGTCCCTACAGGGCAGCCCCGAGAACCACAGGTGTACACCCTGCCCCCATCC
CGGGAGGAGATGACCAAGAACCAGGTCAGCCTGACCTGCCTGGTCAAAGGCTTCTATCCCAGCGACATCGCCGTGGAGTGGGAGAGCAATGGGCAGCCGGAGAACAACTACAAGACCACGCCTCCCGT
GCTGGACTCCGACGGCTCCTTCTTCCTCTATAGCAAGCTCACCGTGGACAAGAGCAGGTGGCAGCAGGGGAACGTCTTCTCATGCTCCGTGATGCATGAGGCTCTGCACAACCACTACACGCAGAAGA
GCCTCTCCCTGTCCCCGGGTAAAGGCGTTGCCATGCCAGGTGCCGAAGATGATGTGGTG

B **PA-scOX40L-His_6**

PA tag-(KpnI)-OX40L-GGGSGGG-OX40L-GGGSGGG-OX40L-(AgeI)-His_6 tag-(STOP)-(BglII)

GGCGTTGCCATGCCAGGTGCCGAAGATGATGTGGTGGGTACCAGCAGCAGCCCCGCGAAAGACCCTCCTATCCAACGCTTGCGCGGCGCCGTCACACGCTGTGAAGACGGCCAACTCTTTATCAGCTC
TTACAAAAATGAGTATCAGACGATGGAAGTCCAGAACAATTCCGTAGTGATAAAGTGTGACGGCCTGTACATTATATATCTGAAAGGCTCCTTCTTCCAGGAGGTGAAGATCGATCTCCACTTTAGGG
AAGACCATAATCCCATCAGCATACCCATGCTGAATGATGGGCGCAGAATAGTGTTTACAGTCGTGGCCAGCCTGGCCTTCAAGGACAAAGTGTACCTCACCGTAAATGCCCCCGACACGCTCTGTGAG
CACCTTCAGATCAATGACGGCGAGCTCATAGTGGTGCAGCTGACACCGGGTTATTGTGCACCCGAAGGCAGCTACCACTCAACCGTGAACCAGGTGCCTCTGGGCGGAGGCTCAGGCGGTGGATCCTC
AAGCCCAGCCAAGGATCCTCCTATCCAACGGCTGAGGGGTGCCGTTACCAGGTGTGAGGATGGCCAGCTCTTCATCAGCTCCTACAAAAATGAGTACCAGACTATGGAGGTGCAAAACAATTCCGTCG
TGATTAAGTGCGATGGTCTCTATATCATCTATCTGAAAGGGAGTTTCTTTCAGGAGGTGAAGATTGACCTTCACTTCAGGGAAGACCACAACCCTATCTCCATTCCTATGCTCAACGATGGCAGAAGA
ATCGTGTTCACCGTCGTCGCTTCCTTGGCCTTTAAAGATAAAGTGTATTTGACTGTGAATGCTCCCGATACCCTGTGCGAACACCTGCAAATCAATGATGGCGAGCTGATTGTGGTGCAGCTTACTCC
CGGTTATTGCGCTCCCGAAGGATCTTACCACTCAACCGTCAATCAAGTTCCTCTGGGAGGAGGATCCGGAGGTGGAAGTTCATCTCCCGCTAAAGATCCGCCAATTCAGAGACTGCGAGGAGCTGTGA
CACGGTGCGAAGACGGGCAGCTGTTCATCTCTTCTTATAAGAACGAGTACCAGACCATGGAGGTGCAGAACAACTCAGTTGTAATTAAGTGCGACGGGCTGTATATCATTTACCTGAAGGGGAGTTTC
TTCCAGGAGGTAAAGATCGATCTGCATTTTCGGGAAGATCATAACCCAATCAGTATTCCGATGCTCAACGATGGGCGGCGAATTGTATTTACAGTCGTCGCGAGTCTTGCATTTAAGGACAAGGTCTA
CCTGACCGTTAACGCACCAGACACATTGTGCGAGCATCTTCAGATTAACGACGGGGAATTGATTGTTGTTCAGCTTACTCCAGGGTACTGCGCACCAGAGGGGTCTTACCATTCTACTGTTAACCAGG
TTCCATTGACCGGTCATCATCACCATCACCATTGAAGATCT

C **PA-sc4-1BBL-His_6**

PA tag-(KpnI)-4-1BBL-GGGSGGG-4-1BBL-GGGSGGG-4-1BBL-(AgeI)-His_6 tag-(STOP)-(BglII)

GGCGTTGCCATGCCAGGTGCCGAAGATGATGTGGTGGGTACCCGAGAGAATAATGCAGACCAGGTCACCCCTGTTTCCCACATTGGCTGCCCCAACACTACACAACAGGGCTCTCCTGTGTTCGCCAA
GCTACTGGCTAAAAACCAAGCATCGTTGTGCAATACAACTCTGAACTGGCACAGCCAAGATGGAGCTGGGAGCTCATACCTATCTCAAGGTCTGAGGTACGAAGAAGACAAAAAGGAGTTGGTGGTAG
ACAGTCCCGGGCTCTACTACGTATTTTTGGAACTGAAGCTCAGTCCAACATTCACAAACACAGGCCACAAGGTGCAGGGCTGGGTCTCTCTTGTTTTGCAAGCAAAGCCTCAGGTAGATGACTTTGAC
AACTTGGCCCTGACAGTGGAACTGTTCCCTTGCTCCATGGAGAACAAGTTAGTGGACCGTTCCTGGAGTCAACTGTTGCTCCTGAAGGCTGGCCACCGCCTCAGTGTGGGTCTGAGGGCTTATCTGCA
TGGAGCCCAGGATGCATACAGAGACTGGGAGCTGTCTTATCCCAACACCACCAGCTTTGGACTCTTTCTTGTGAAACCCGACAACCCATGGGAAGGTGGAGGATCCGGTGGAGGTCGAGAGAATAATG
CAGACCAGGTCACCCCTGTTTCCCACATTGGCTGCCCCAACACTACACAACAGGGCTCTCCTGTGTTCGCCAAGCTACTGGCTAAAAACCAAGCATCGTTGTGCAATACAACTCTGAACTGGCACAGC
CAAGATGGAGCTGGGAGCTCATACCTATCTCAAGGTCTGAGGTACGAAGAAGACAAAAAGGAGTTGGTGGTAGACAGTCCCGGGCTCTACTACGTATTTTTGGAACTGAAGCTCAGTCCAACATTCAC
AAACACAGGCCACAAGGTGCAGGGCTGGGTCTCTCTTGTTTTGCAAGCAAAGCCTCAGGTAGATGACTTTGACAACTTGGCCCTGACAGTGGAACTGTTCCCTTGCTCCATGGAGAACAAGTTAGTGG
ACCGTTCCTGGAGTCAACTGTTGCTCCTGAAGGCTGGCCACCGCCTCAGTGTGGGTCTGAGGGCTTATCTGCATGGAGCCCAGGATGCATACAGAGACTGGGAGCTGTCTTATCCCAACACCACCAGC
TTTGGACTCTTTCTTGTGAAACCCGACAACCCATGGGAAGGAGGCGGTTCCGGAGGTGGGCGAGAGAATAATGCAGACCAGGTCACCCCTGTTTCCCACATTGGCTGCCCCAACACTACACAACAGGG
CTCTCCTGTGTTCGCCAAGCTACTGGCTAAAAACCAAGCATCGTTGTGCAATACAACTCTGAACTGGCACAGCCAAGATGGAGCTGGGAGCTCATACCTATCTCAAGGTCTGAGGTACGAAGAAGACA
AAAAGGAGTTGGTGGTAGACAGTCCCGGGCTCTACTACGTATTTTTGGAACTGAAGCTCAGTCCAACATTCACAAACACAGGCCACAAGGTGCAGGGCTGGGTCTCTCTTGTTTTGCAAGCAAAGCCT
CAGGTAGATGACTTTGACAACTTGGCCCTGACAGTGGAACTGTTCCCTTGCTCCATGGAGAACAAGTTAGTGGACCGTTCCTGGAGTCAACTGTTGCTCCTGAAGGCTGGCCACCGCCTCAGTGTGGG
TCTGAGGGCTTATCTGCATGGAGCCCAGGATGCATACAGAGACTGGGAGCTGTCTTATCCCAACACCACCAGCTTTGGACTCTTTCTTGTGAAACCCGACAACCCATGGGAAACCGGTCATCATCACC
ATCACCATTGAAGATCT

D **PA-scCD70-His_6**

PA tag-(KpnI)-CD70-GGGSGGG-CD70-GGGSGGG-CD70-(AgeI)-His_6 tag-(STOP)-(BglII)

GGCGTTGCCATGCCAGGTGCCGAAGATGATGTGGTGGGTACCAGTAAGCAGCAACAGAGGCTGCTGGAGCACCCTGAGCCGCACACAGCTGAGTTACAGCTGAATCTCACAGTTCCTCGGAAGGACCC
CACACTGCGCTGGGGAGCAGGCCCAGCCTTGGGAAGGTCCTTCACACACGGACCAGAGCTGGAGGAGGGCCATCTGCGTATCCATCAAGATGGCCTCTACAGGCTGCATATCCAGGTGACACTGGCCA
ACTGCTCTTCCCCAGGCAGCACCCTGCAGCACAGGGCCACCCTGGCTGTGGGCATCTGCTCCCCCGCTGCGCACGGCATCAGCTTGCTGCGTGGGCGCTTTGGACAGGACTGTACAGTGGCATTACAG
CGCCTGACATACCTGGTCCACGGAGATGTCCTCTGTACCAACCTCACCCTGCCTCTGCTGCCGTCCCGCAACGCTGATGAGACCTTCTTTGGAGTTCAGTGGATATGCCCTGGTGGAGGATCCGGTGG
AGGTAGTAAGCAGCAACAGAGGCTGCTGGAGCACCCTGAGCCGCACACAGCTGAGTTACAGCTGAATCTCACAGTTCCTCGGAAGGACCCCACACTGCGCTGGGGAGCAGGCCCAGCCTTGGGAAGGT
CCTTCACACACGGACCAGAGCTGGAGGAGGGCCATCTGCGTATCCATCAAGATGGCCTCTACAGGCTGCATATCCAGGTGACACTGGCCAACTGCTCTTCCCCAGGCAGCACCCTGCAGCACAGGGCC
ACCCTGGCTGTGGGCATCTGCTCCCCCGCTGCGCACGGCATCAGCTTGCTGCGTGGGCGCTTTGGACAGGACTGTACAGTGGCATTACAGCGCCTGACATACCTGGTCCACGGAGATGTCCTCTGTAC
CAACCTCACCCTGCCTCTGCTGCCGTCCCGCAACGCTGATGAGACCTTCTTTGGAGTTCAGTGGATATGCCCTGGAGGCGGTTCCGGAGGTGGGAGTAAGCAGCAACAGAGGCTGCTGGAGCACCCTG
AGCCGCACACAGCTGAGTTACAGCTGAATCTCACAGTTCCTCGGAAGGACCCCACACTGCGCTGGGGAGCAGGCCCAGCCTTGGGAAGGTCCTTCACACACGGACCAGAGCTGGAGGAGGGCCATCTG
CGTATCCATCAAGATGGCCTCTACAGGCTGCATATCCAGGTGACACTGGCCAACTGCTCTTCCCCAGGCAGCACCCTGCAGCACAGGGCCACCCTGGCTGTGGGCATCTGCTCCCCCGCTGCGCACGG
CATCAGCTTGCTGCGTGGGCGCTTTGGACAGGACTGTACAGTGGCATTACAGCGCCTGACATACCTGGTCCACGGAGATGTCCTCTGTACCAACCTCACCCTGCCTCTGCTGCCGTCCCGCAACGCTG
ATGAGACCTTCTTTGGAGTTCAGTGGATATGCCCTACCGGTCATCATCACCATCACCATTGAAGATCT

E **PA-scGITRL-His_6**

PA tag-(KpnI)-GITRL-GGGSGGG-GITRL-GGGSGGG-GITRL-(AgeI)-His_6 tag-(STOP)-(BglII)

GGCGTTGCCATGCCAGGTGCCGAAGATGATGTGGTGGGTACCACTTCACTCAAGCCAACTGCCATCGAGTCCTGCATGGTTAAGTTTGAACTATCATCCTCAAAATGGCACATGACATCTCCCAAACC
TCACTGTGTGAATACGACATCTGATGGGAAGCTGAAGATACTGCAGAGTGGCACATATTTAATCTACGGCCAAGTGATTCCTGTGGATAAGAAATACATAAAAGACAATGCCCCCTTCGTAGTACAGA
TATATAAAAAGAATGATGTCCTACAAACTCTAATGAATGATTTTCAAATCTTGCCTATAGGAGGGGTTTATGAACTGCATGCTGGAGATAACATATATCTGAAGTTCAACTCTAAAGACCATATTCAG
AAAAATAACACATACTGGGGGATCATCTTAATGCCTGATCTACCATTCATCTCTGGTGGAGGATCCGGTGGAGGTACTTCACTCAAGCCAACTGCCATCGAGTCCTGCATGGTTAAGTTTGAACTATC
ATCCTCAAAATGGCACATGACATCTCCCAAACCTCACTGTGTGAATACGACATCTGATGGGAAGCTGAAGATACTGCAGAGTGGCACATATTTAATCTACGGCCAAGTGATTCCTGTGGATAAGAAAT
ACATAAAAGACAATGCCCCCTTCGTAGTACAGATATATAAAAAGAATGATGTCCTACAAACTCTAATGAATGATTTTCAAATCTTGCCTATAGGAGGGGTTTATGAACTGCATGCTGGAGATAACATA
TATCTGAAGTTCAACTCTAAAGACCATATTCAGAAAAATAACACATACTGGGGGATCATCTTAATGCCTGATCTACCATTCATCTCTGGAGGCGGTTCCGGAGGTGGGACTTCACTCAAGCCAACTGC
CATCGAGTCCTGCATGGTTAAGTTTGAACTATCATCCTCAAAATGGCACATGACATCTCCCAAACCTCACTGTGTGAATACGACATCTGATGGGAAGCTGAAGATACTGCAGAGTGGCACATATTTAA
TCTACGGCCAAGTGATTCCTGTGGATAAGAAATACATAAAAGACAATGCCCCCTTCGTAGTACAGATATATAAAAAGAATGATGTCCTACAAACTCTAATGAATGATTTTCAAATCTTGCCTATAGGA
GGGGTTTATGAACTGCATGCTGGAGATAACATATATCTGAAGTTCAACTCTAAAGACCATATTCAGAAAAATAACACATACTGGGGGATCATCTTAATGCCTGATCTACCATTCATCTCTACCGGTCA
TCATCACCATCACCATTGAAGATCT

Figure S1. Nucleotide sequences of Fc–scTNFL proteins.

Figure S2

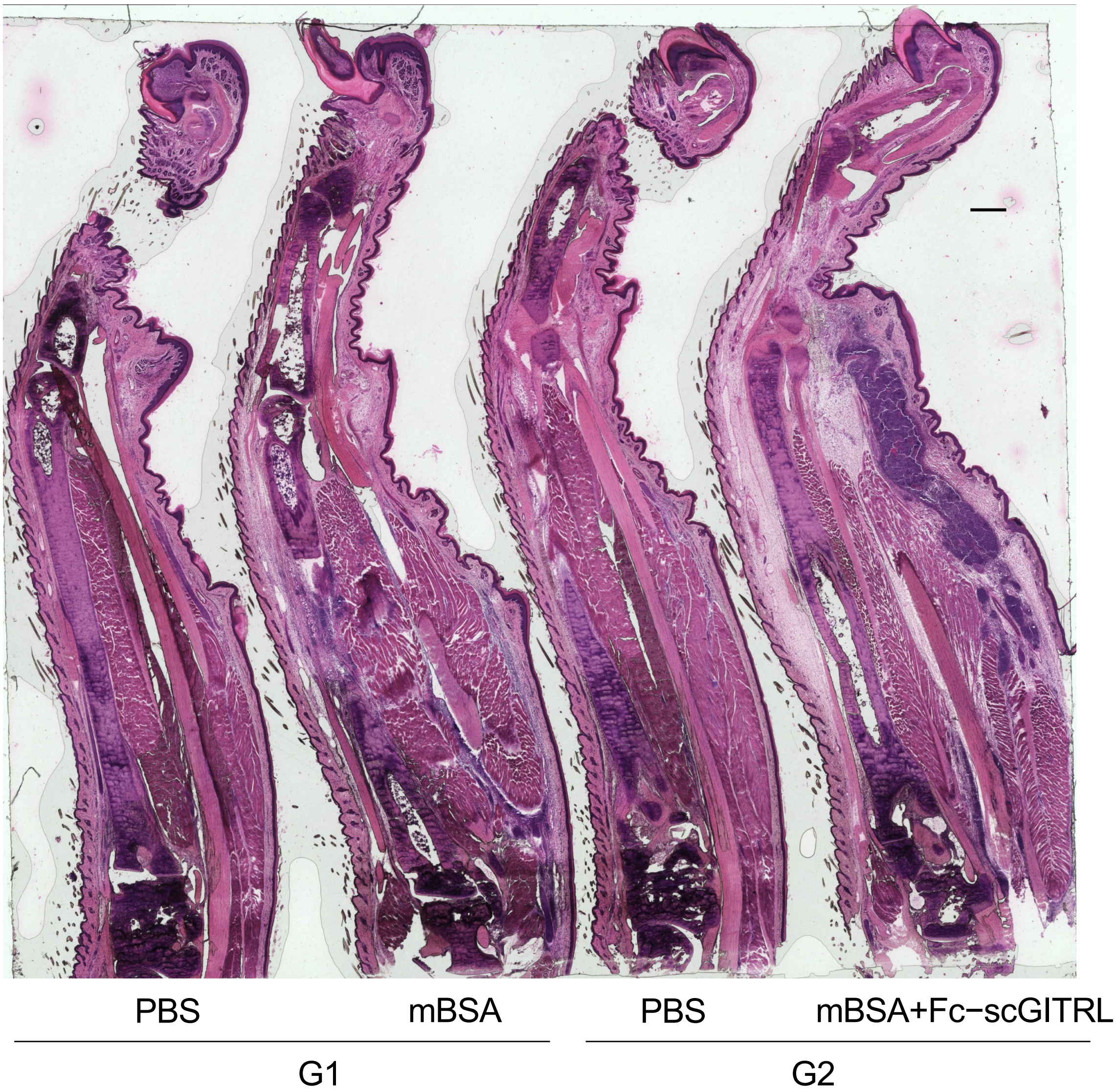

Figure S2. Challenge injection of Fc−scGITRL augments DTH response.
Hematoxylin-eosin staining of footpad sections at day 8. Scale bar, 500 μm.
